# Supplementary figures and images for: Hello, is that me you are looking for? A re-examination of the role of the DMN in social and self relevant aspects of off-task thought
Source: PLoS One. 2019 Nov 7;14(11):e0216182. doi: 10.1371/journal.pone.0216182 (PMC6837379; doi:10.1371/journal.pone.0216182)

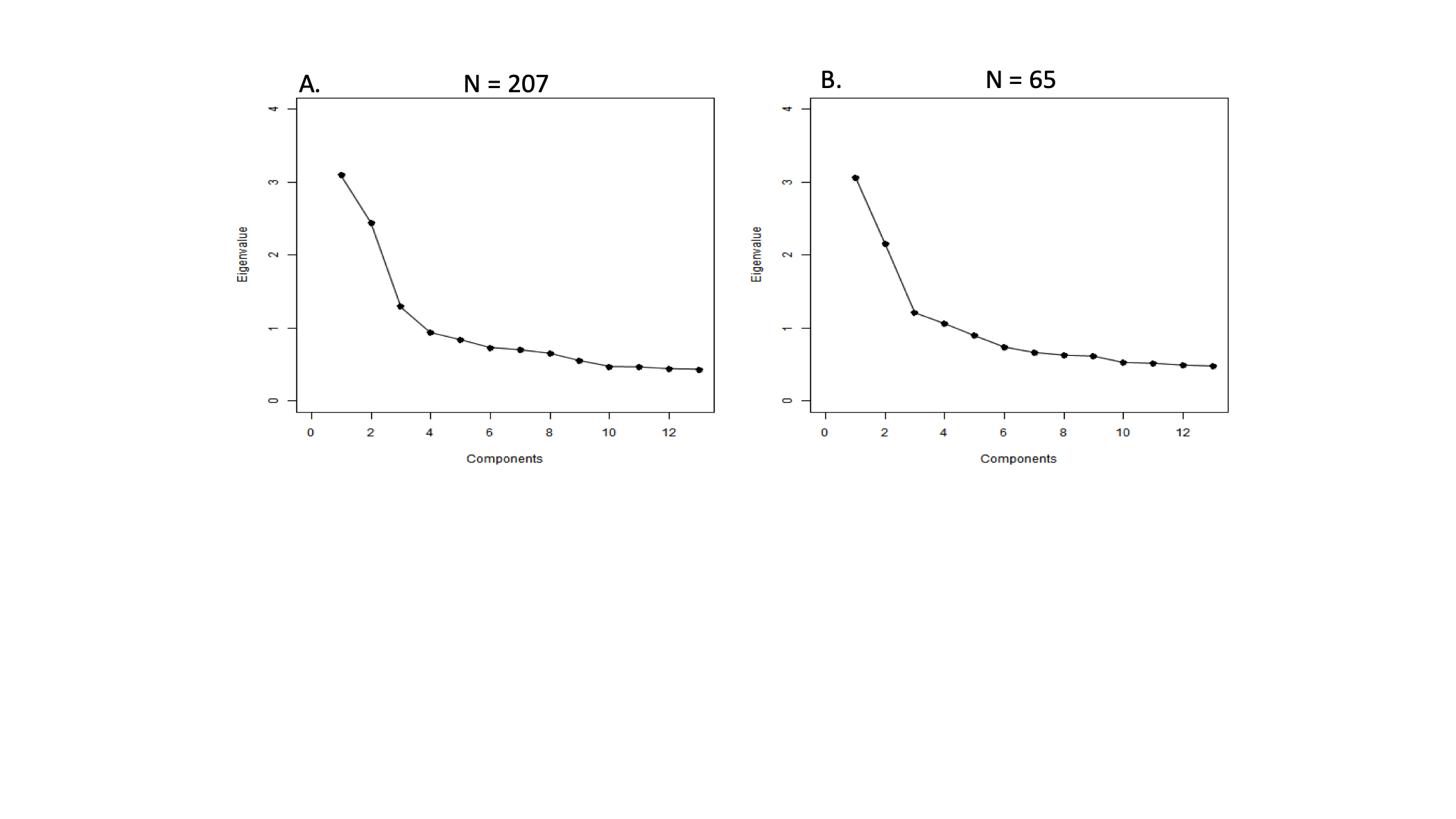

Supplement: S1 Fig — Scree plot describing the decomposition of experience sampling data in the lab for all 207 participants (A) and for a subset of these (N = 65) who took part in the self-reference task (B). In both decompositions the first four components revealed a significant positive correlation (PCA1 –Detail; r = .69, p = .009; PCA2 –Off-task; r = .72, p = .005; PCA3 –Modality; r = .94, p < .001; PCA4 –Emotion; r = -.65, p = .02). (TIFF) [file pone.0216182.s001.tiff]

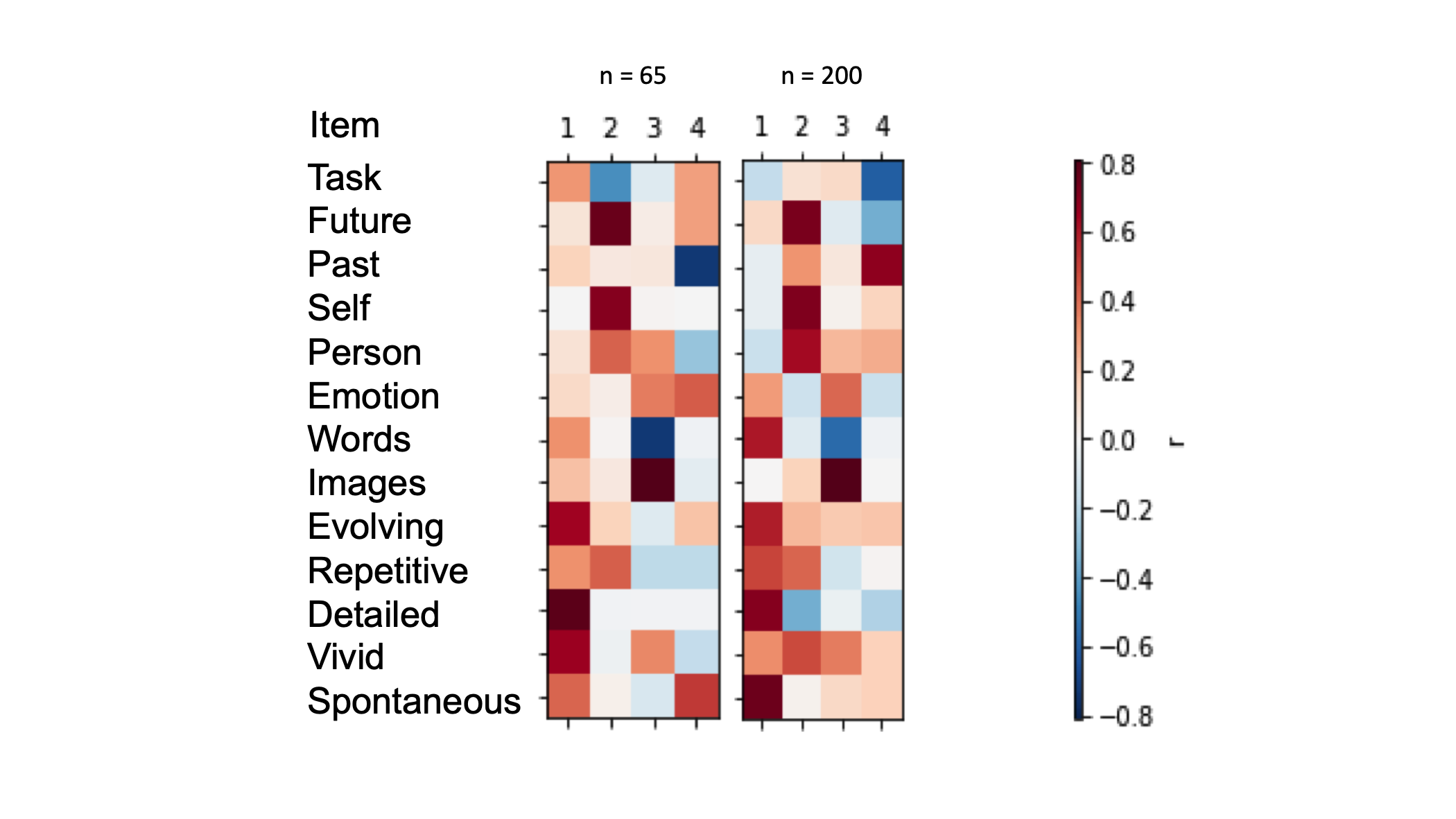

Supplement: S2 Fig — Heat maps describing the decomposition of experience sampling data in the lab for all 207 participants (left) and for a subset of these (N = 65) who took part in the self-reference task (right). Correlations between the maps revealed a significant relationship between each component across the two datasets (PCA1 –Detail; r = .69, p = .009; PCA2 –Off-task; r = .72, p = .005; PCA3 –Modality; r = .94, p < .001; PCA4 –Emotion; r = -.65, p = .02). (TIFF) [file pone.0216182.s002.tiff]

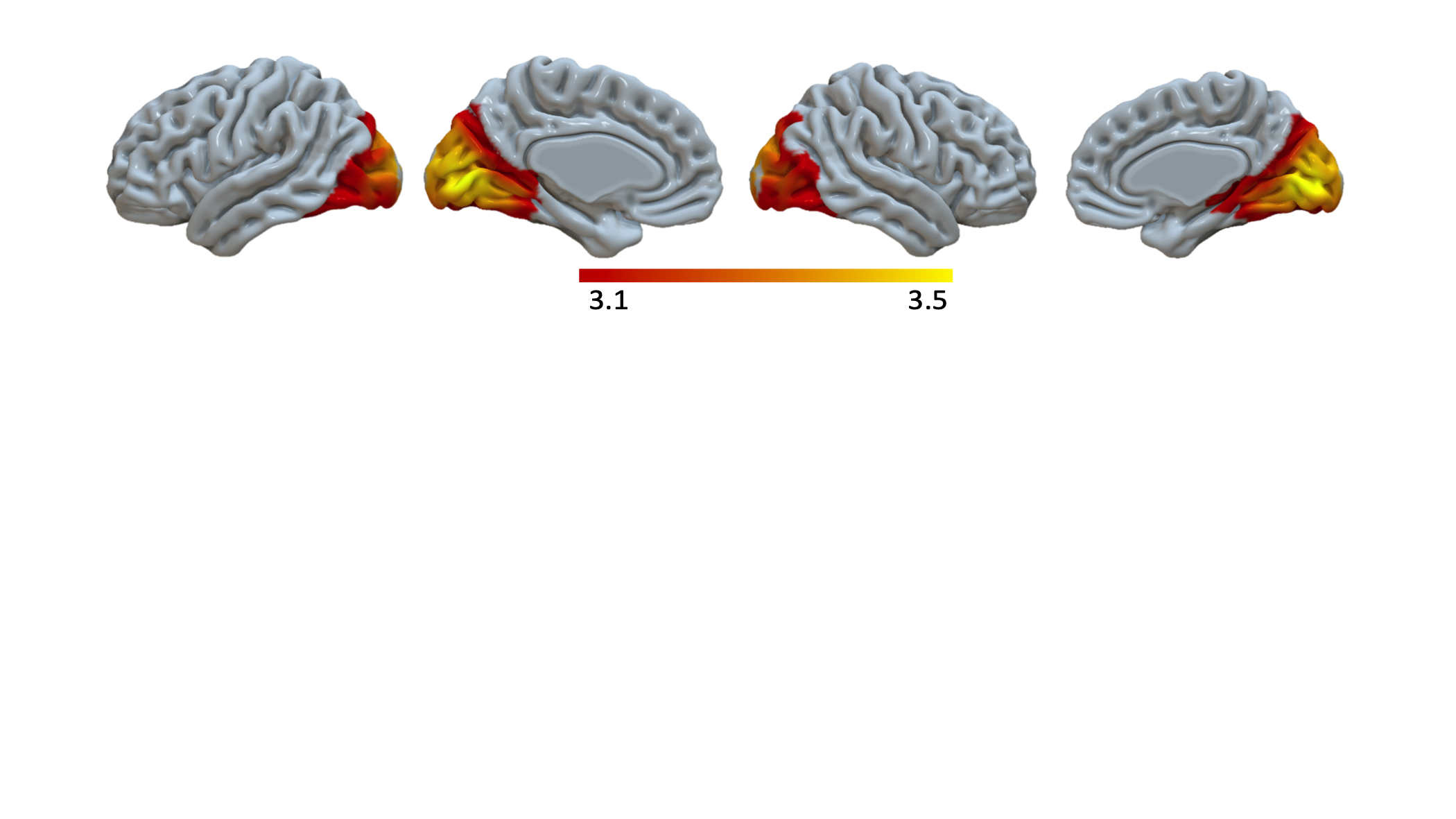

Supplement: S3 Fig — (TIFF) [file pone.0216182.s003.tiff]

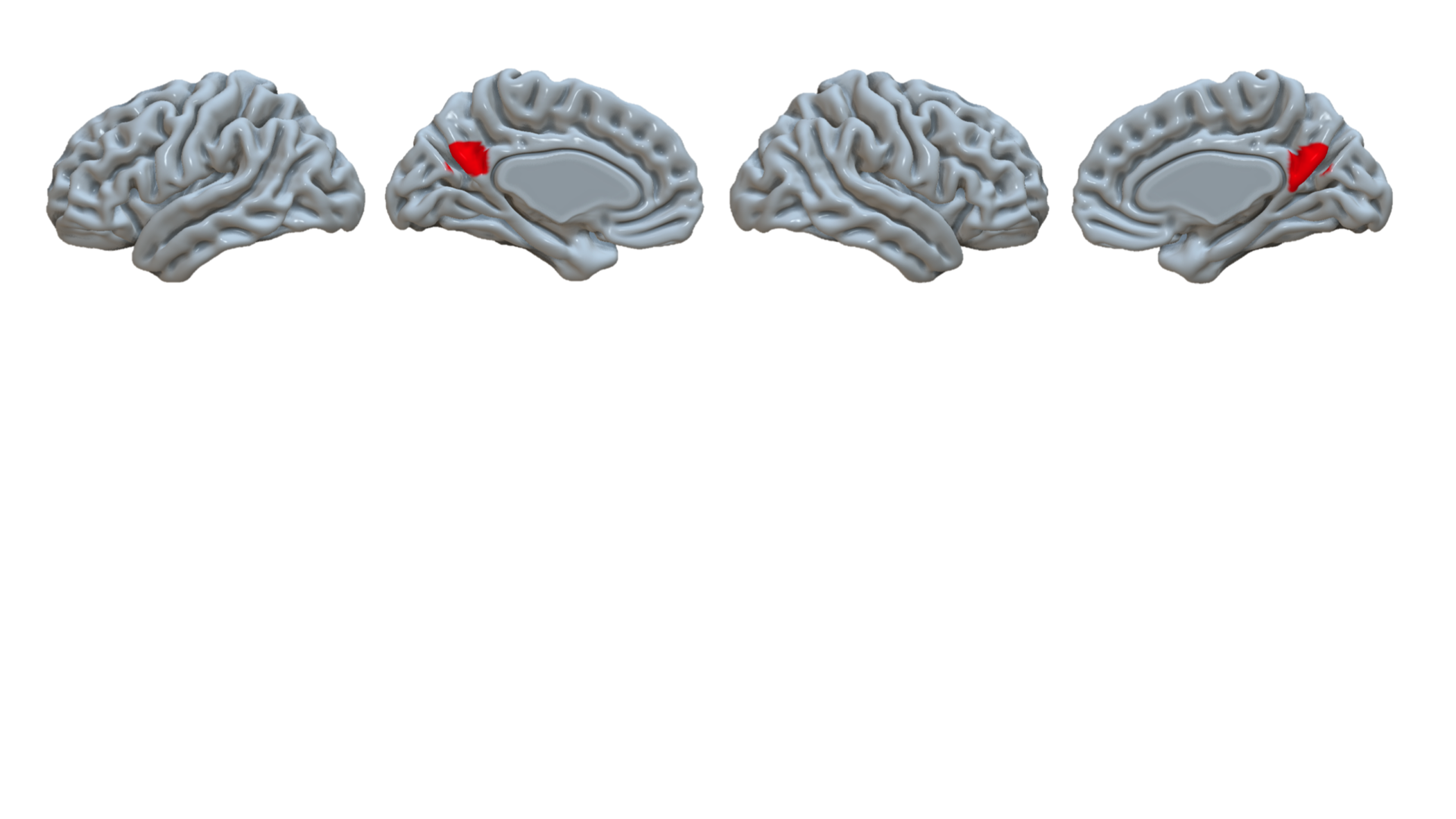

Supplement: S4 Fig — The spatial maps are thresholded at Z = 3.1 and are controlled for multiple comparisons (p < .05, FWE). (TIFF) [file pone.0216182.s004.tiff]

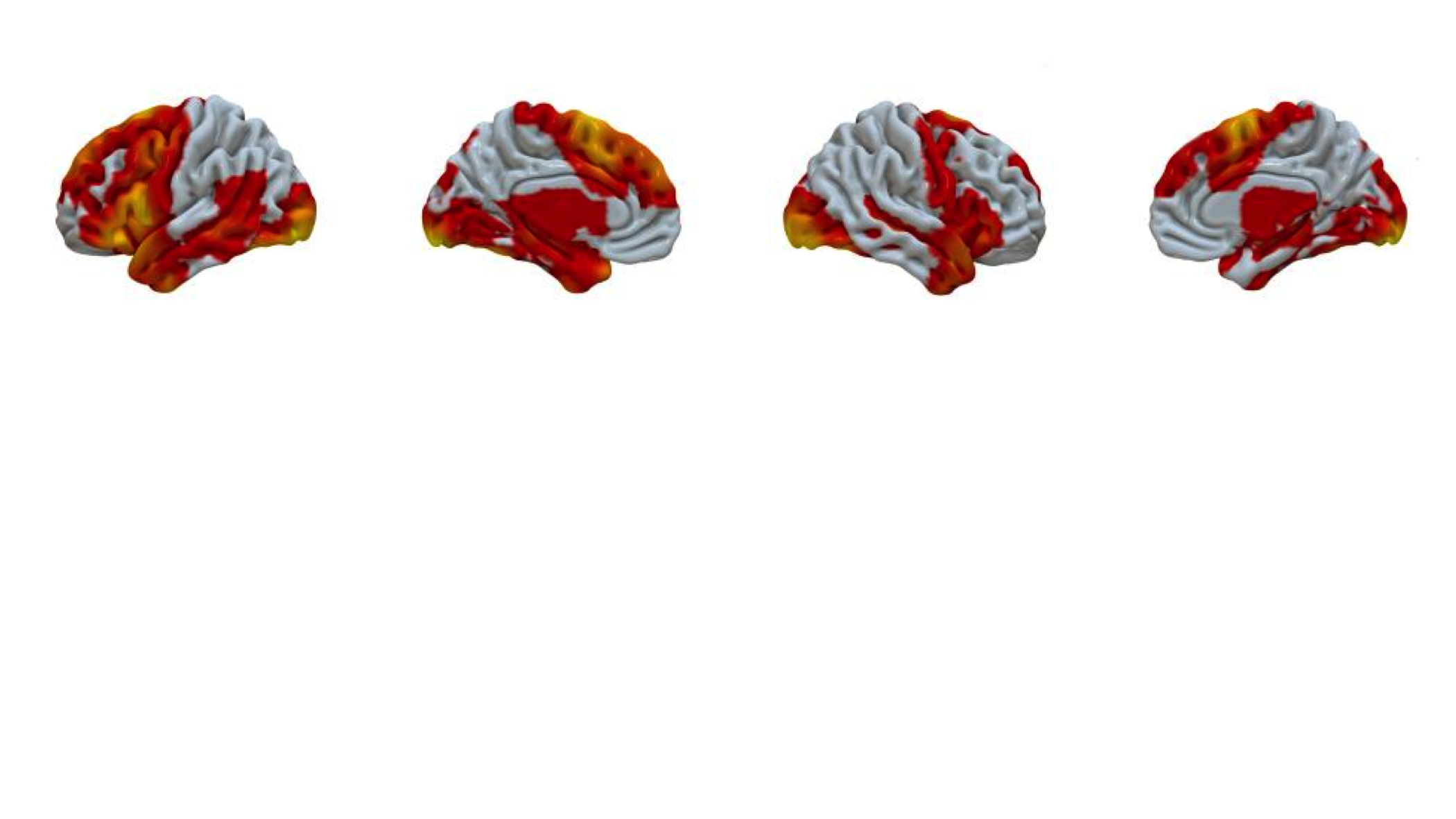

Supplement: S5 Fig — All reported clusters were corrected for multiple comparisons using the Family-Wise Error (FWE) detection technique at the .05 level of significance (uncorrected at the voxel-level, .001 level of significance). (TIFF) [file pone.0216182.s005.tiff]
