# Supplementary material for: Hello, is that me you are looking for? A re-examination of the role of the DMN in social and self relevant aspects of off-task thought
Source: PLoS One. 2019 Nov 7;14(11):e0216182. doi: 10.1371/journal.pone.0216182 (PMC6837379; doi:10.1371/journal.pone.0216182)
Supplement: S1 Table — (DOCX) [file pone.0216182.s006.docx]

|  |  |  |  |  |  |  |
| --- | --- | --- | --- | --- | --- | --- |
| Contrast | Cluster | Brain Regions | MNI co-ordinates | Voxels | Z-value | p-value |
| Self > Other | 1 | Medial prefrontal cortex | 0 40 0 | 2694 | 6.70 | < .001 |
|  | 2 | L. Precuneous | -22 -52 6 | 1704 | 5.40 | < .001 |
|  | 3 | R. Occipital fusiform gyrus | 20 -88 -14 | 1002 | 4.25 | <.001 |
|  | 4 | Anterior cingulate gyrus | -2 -18 32 | 259 | 5.01 | .029 |
| Other > Self | 1 | R. Lateral occipital cortex, superior | 52 -68 20 | 1494 | 6.35 | < .001 |
|  | 2 | Precuneous | 4 -66 32 | 1058 | 6.53 | < .001 |
|  | 3 | L. Lateral Occipital cortex, superior | -48 -66 22 | 673 | 4.53 | < .001 |
|  | 4 | L. Middle temporal gyrus, anterior | -56 -12 -18 | 561 | 6.04 | < .001 |
|  | 5 | R. Middle temporal gyrus, anterior | 60 -2 -28 | 526 | 5.23 | <.001 |
|  | 6 | R. Inferior temporooccipital cortex | 66 -46 -14 | 249 | 4.46 | .033 |

S1 Table. Location of peak coordinates from the univariate contrasts of Self > Other and Other > Self.
