# Supplementary material for: Hello, is that me you are looking for? A re-examination of the role of the DMN in social and self relevant aspects of off-task thought
Source: PLoS One. 2019 Nov 7;14(11):e0216182. doi: 10.1371/journal.pone.0216182 (PMC6837379; doi:10.1371/journal.pone.0216182)
Supplement: S2 Table — (DOCX) [file pone.0216182.s007.docx]

S2 Table. Location of peak coordinates from the univariate contrasts of Self and Other regressed against the PCA loadings from the MDES.

|  |  |  |  |  |  |  |  |
| --- | --- | --- | --- | --- | --- | --- | --- |
| Contrast | PCA | Cluster | Brain Regions | MNI co-ordinates | Voxels | Z-value | p-value |
| Self | 1 - Detail | 1 | Caudal Posterior Cingulate Cortex | 0 -58 24 | 254 | 3.89 | .036 |
| Other | 1 - Detail | 1 | R. Intracalcarine cortex/lingual gyrus | 18 -80 4 | 342 | 4.18 | .005 |
